# Acute biomechanical responses to stroboscopic vision during change-of-direction tasks in elite rugby athletes

**DOI:** 10.3389/fphys.2026.1900961

**Published:** 2026-09-08

**Authors:** Lehai Lin, Hongji Chen, Xu Huang, Qi Zhong, Shuyu Xue, Tianhe Li, Yapu Liang

**Affiliations:** 1 School of Strength and Conditioning Training, Beijing Sport University, Beijing, China; 2 School of Sports Medicine and Rehabilitation, Beijing Sport University, Beijing, China; 3 Sports Coaching College, Beijing Sport University, Beijing, China

**Keywords:** biomechanical, change of direction, intermittent visual occlusion, rugby, side-cutting manoeuver, stroboscopic vision

## Abstract

**Introduction:**

Stroboscopic vision (SV), characterized by intermittent visual occlusion, has been proposed as a novel perceptual stimulus to challenge sensorimotor integration in sport. This study aimed to investigate the acute effects of SV on lower-limb biomechanics during rugby-specific side-cutting manoeuvers.

**Methods:**

Twenty-one elite male rugby athletes performed side-cutting tasks under full vision (FV) and SV conditions, both with and without ball-carrying, using a randomized crossover design. Three-dimensional kinematics and ground reaction forces (GRF) were recorded via an eight-camera motion capture system and a force platform. Paired-samples t-tests or Wilcoxon signed-rank tests were used according to the distribution of paired differences, and effect sizes were calculated as Cohen’s *d* or *r*, respectively. Statistical significance was set at p< 0.05.

**Results:**

Under SV, non-ball-carrying side-cutting showed significantly greater peak vertical GRF (p = 0.033, d = 0.614), more negative hip moment (p< 0.001, r = 0.808), greater knee moment (p = 0.006, r = 0.693), and greater peak knee joint power (p< 0.001, r = 0.869). During ball-carrying side-cutting, SV was associated with greater peak vertical GRF (p = 0.003, r = 0.726), more negative hip moment (p< 0.001, r = 0.876), greater knee moment (p = 0.006, d = 0.785), and greater peak knee joint power (p = 0.048, r = 0.527). No significant effects of SV were observed for ankle joint moments or power.

**Conclusion:**

Acute SV exposure was associated with altered lower-limb loading and joint mechanics during rugby-related side-cutting, with the most consistent changes observed in vertical GRF and hip and knee joint kinetics. Longitudinal studies incorporating neuromuscular, perceptual, and performance measures are needed to determine whether repeated SV exposure produces transferable training adaptations relevant to rugby performance.

## 1 Introduction

Rugby is characterized by a chaotic and fast-changing environment, where athletes must continuously adapt to opponents’ movements, unpredictable ball trajectories, and evolving spatial configurations. Effective performance in such contexts relies on the central nervous system’s (CNS) ability to rapidly integrate and prioritize information from multiple sensory systems to guide timely and accurate motor output (Chow et al., 2017; Millard et al., 2023). Among these systems, vision provides the dominant stream of information for perception–action coupling, supporting the detection of defensive alignments, recognition of open space, and prediction of collision risks (Oppici and Panchuk, 2022; Millard et al., 2023; Dixon et al., 2025). During high-velocity, multiplanar tasks such as evasive side-stepping or rapid cutting, players are required to interpret these visual cues within milliseconds to select an optimal running line, adjust body orientation, and coordinate lower-limb mechanics to maintain balance and momentum (Rikken et al., 2024; Xu et al., 2025). Visual input not only informs the initiation and direction of movement but also modulates preparatory muscle activity and contact strategies, thereby shaping both anticipatory and reactive motor control (Smith and Fisher, 2018; Rolley et al., 2023; Berg, 2024). When visual information is degraded or delayed, movement execution and lower-limb biomechanical responses may be altered, increasing the likelihood of suboptimal movement execution or destabilization—an especially critical risk under the time-pressure and collision demands inherent to rugby match play (Wilke et al., 2021; Xiong and Song, 2024; Pourhashemi et al., 2025).

Stroboscopic vision (SV) entails the deliberate disruption of visual input using eyewear that alternates rhythmically between transparent and opaque phases, thus periodically restricting visual access (Smith and Mitroff, 2012; Das et al., 2023; Li et al., 2024a). Through these intermittent occlusions, the availability and reliability of visual information are reduced. Previous theoretical and experimental research has suggested that reduced visual reliability may be accompanied by changes in the relative reliance on visual, proprioceptive, and vestibular information (Jang et al., 2022; Symeonidou and Ferris, 2022; Harper et al., 2024). This sensory reweighting mechanism is thought to challenge perceptual stability and foster more flexible motor control adaptations (Chung and Barnett-Cowan, 2023; Dewan et al., 2023). While SV has been widely examined in sports such as basketball and soccer (Wilkins et al., 2018; Wilkins and Appelbaum, 2020; Luo et al., 2025), its specific application and benefits in rugby remain insufficiently understood. Effective rugby performance relies heavily on perceptual–motor adaptability within dynamic, collision-intensive contexts—particularly during evasive side-stepping, ball handling under pressure, and aerial contests (Oppici and Panchuk, 2022; van Paridon et al., 2022; Millard et al., 2023).

Rugby-specific studies have consistently emphasized the importance of visual–perceptual and decision-making skills for successful performance (van Paridon et al., 2022). Expert rugby players exhibit more functional visual search strategies during attacking and defensive scenarios, such as reading opponents’ running lines or anticipating tackle intentions (Gabbett et al., 2011). Moreover, decision-making under defensive pressure has been identified as a key differentiator between successful and less successful ball carriers, with greater visual–cognitive load directly influencing movement execution and tactical outcomes (Wilkins and Appelbaum, 2020; Turek et al., 2024). Nevertheless, evidence from multidirectional and unanticipated movement tasks suggests that visual perturbations or distractions can alter lower-limb biomechanics, increasing ground reaction forces (GRF) and modifying muscle activation timing, thereby influencing both performance and injury risk (Wilke et al., 2021). These findings suggest that visual perturbation may not only affect decision-making but also the mechanical robustness of movements critical to injury prevention and performance in rugby (Xu et al., 2025).

Most prior studies have addressed general agility or ball-handling tasks, with minimal attention to rugby-related side-cutting actions requiring the integration of perceptual and motor processes. In particular, performing side-cutting manoeuvers while carrying a ball introduces an additional task constraint because athletes must maintain ball possession while completing the prescribed directional-change movement. Intermittent visual occlusion may therefore alter lower-limb biomechanics during this task. Accordingly, the present study aimed to examine the acute effects of SV compared to full vision (FV) on lower-limb biomechanical variables during side-cutting manoeuvers, both with and without ball-carrying, in elite rugby athletes. It was hypothesized that: compared with FV, SV would alter lower-limb biomechanics during rugby-related side-cutting performed both with and without ball-carrying.

## 2 Materials and methods

### 2.1 Ethical approval and participants

Twenty-one elite male rugby athletes were recruited for this investigation (age: 19.67 ± 1.49 years; height: 180.33 ± 5.93 cm; body mass: 72.71 ± 8.14 kg; training experience: 7.05 ± 1.36 years; all right-leg dominant). Participants were male rugby athletes recruited from provincial-team rugby teams and university varsity rugby teams in China. During the four weeks preceding the experiment, participants completed an average of 10.6 ± 2.8 h per week of rugby-specific training and strength and conditioning training. The participants’ characteristics are presented in **Table 1**.

**Table 1:** Participant characteristics.

Variable	Mean ± SD or N
Sample Size	21
Gender	Male
Age (yrs)	19.67 ± 1.49
Height (cm)	180.33 ± 5.93
Body Mass (kg)	72.71 ± 8.14
Training Experience (yrs)	7.05 ± 1.36

The results are presented as mean ± SD.

Eligibility criteria for inclusion comprised the following: (a) aged between 18 and 30 years; (b) currently competed for provincial-level rugby teams or university varsity rugby teams in China (McKay et al., 2022); (c) have a minimum of five years of structured rugby training experience; (d) possess normal or corrected-to-normal vision; (e) be free from any neuromuscular or vestibular disorders; (f) to standardize the side-cutting task, the left leg was designated as the plant leg for all trials. Participants were excluded if they: (a) had sustained any lower limb musculoskeletal injury or undergone surgery within the previous six months; (b) reported current pain or discomfort during training or testing sessions; (c) had been diagnosed with any neurological or vestibular disorder; (d) were known to be sensitive or intolerant to stroboscopic visual stimuli; (e) presented with visual impairment that could not be corrected through standard optical means (e.g., contact lenses or glasses); (f) had a history of concussion or epilepsy; or (g) reported recent use (within the past seven days) of stimulants or performance-enhancing substances. All participants granted informed consent before participating in the study.

The study was conducted according to the guidelines of the Declaration of Helsinki and approved by the Sports Science Experiment Ethics Committee of Beijing Sport University (2025324H). This study was prospectively registered with the Chinese Clinical Trial Registry (ChiCTR), which is a primary registry in the WHO International Clinical Trials Registry Platform. The registration number is ChiCTR2500108470, and the registration website is http://www.chictr.org.cn/.

### 2.2 Design

**Figure 1:**
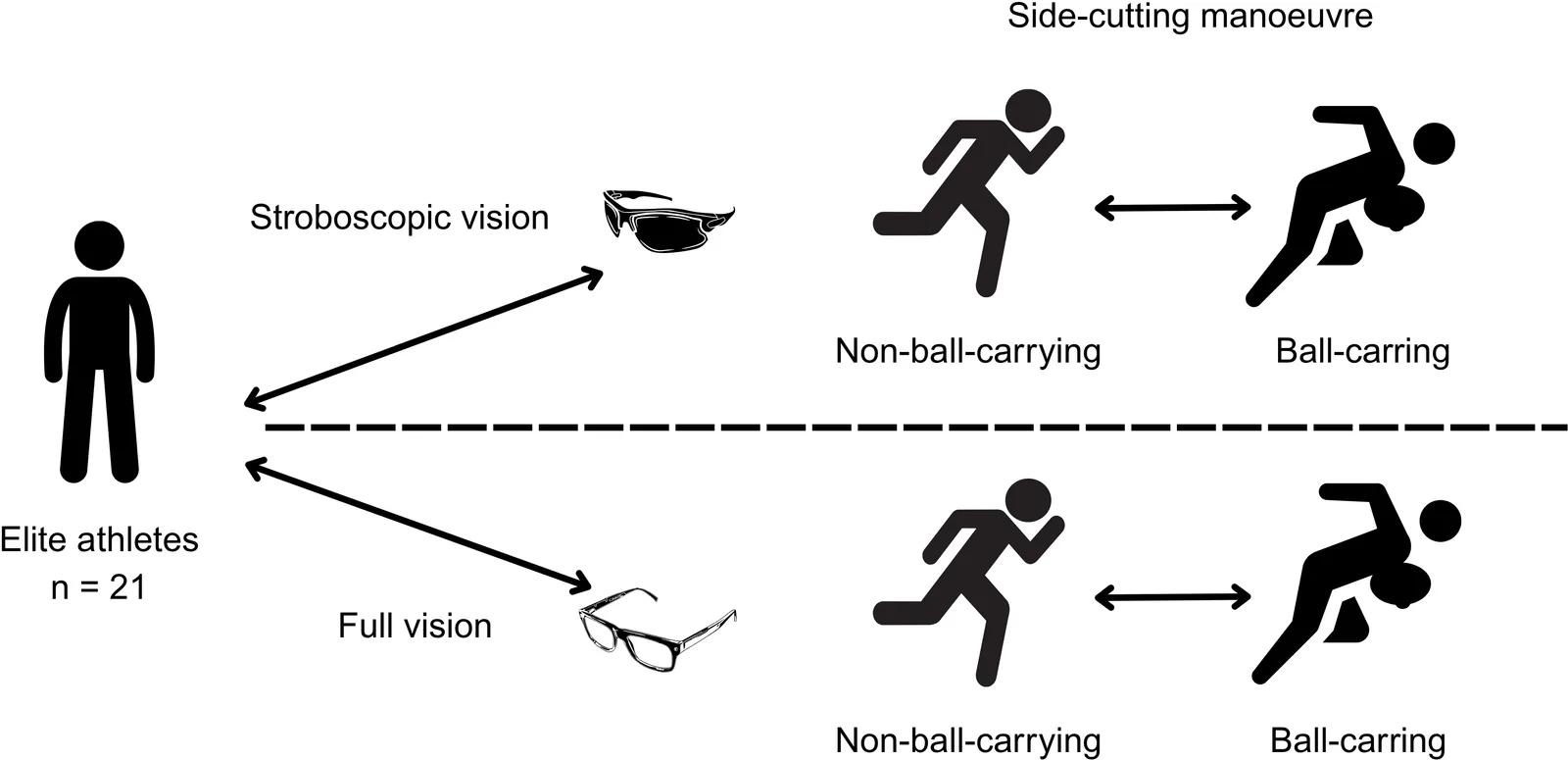
Schematic illustration of the experimental procedure.

**Figure 2:**
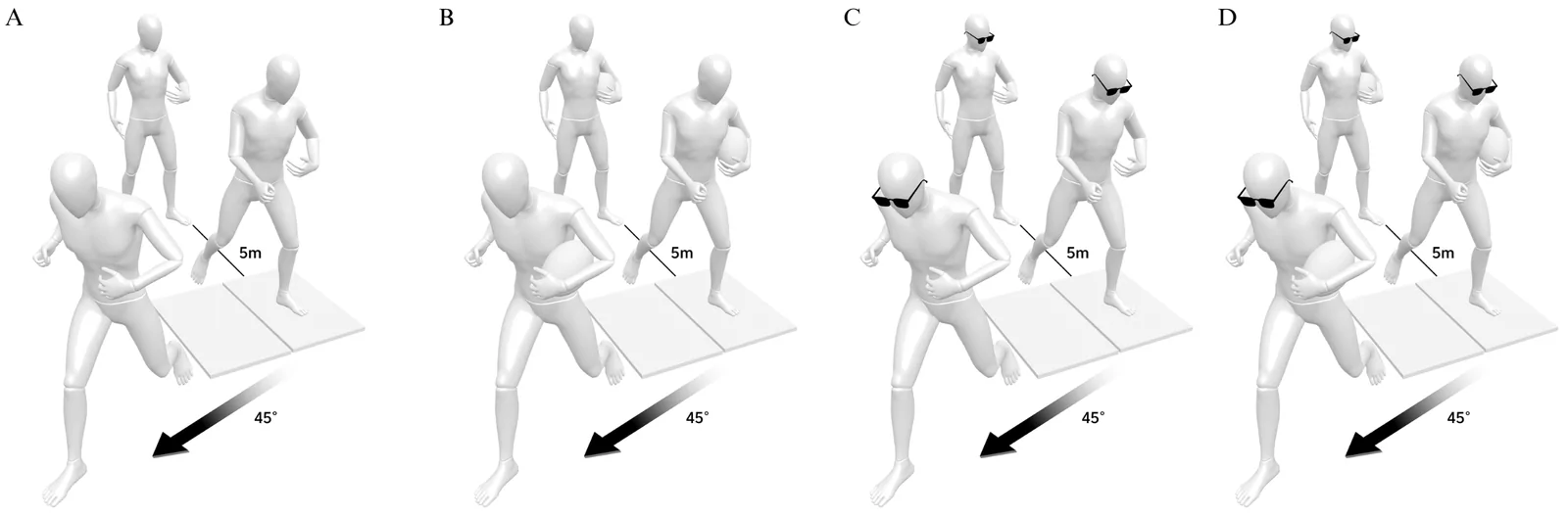
Schematic illustration of the side-cutting manoeuver. **(A)** non-ball-carrying side-cutting under full vision (FV); **(B)** ball-carrying side-cutting under FV; **(C)** non-ball-carrying side-cutting under stroboscopic vision (SV); and **(D)** ball-carrying side-cutting under SV.

A randomized crossover design was employed in this study to investigate the biomechanical effects of visual conditions on side-cut manoeuver performance in elite male rugby athletes. Participants were randomly assigned to begin with either the FV or SV condition. Under each visual condition, participants performed side-cutting manoeuvers in both non-ball-carrying and ball-carrying tasks, resulting in four experimental conditions in total. The order of conditions was randomized across participants. Each trial was repeated three times. The experiment was conducted at the School of Strength and Conditioning Training, Beijing Sport University. Investigators responsible for administering the interventions were necessarily aware of the condition being delivered. Data files were coded before statistical analysis so that the analyst was blinded to the visual and task conditions. This approach was adopted to minimize potential assessment and analysis bias. The experimental procedure and trial schematic are illustrated in **Figures 1**, **2**.

### 2.3 Protocol

Data were collected using a three-dimensional motion capture system and a force platform, focusing on the left-leg support phase. Three-dimensional lower-limb kinematic data were recorded using an eight-camera Qualisys motion analysis system (Qualisys AB, Gothenburg, Sweden) operating at a sampling frequency of 200 Hz. A total of 22 reflective markers were attached to anatomical landmarks of the pelvis and lower limbs based on an extended version of the Helen Hayes model (Davis III et al., 1991). The GRF data were synchronously acquired at 1000 Hz using a Kistler force platform (Kistler Instruments, Winterthur, Switzerland).

Prior to testing, participants completed a 10-minute standardized warm-up routine. To ensure consistency in the direction of movement, adhesive tape markers were placed on the ground at a 45° angle to the right side. A 5-metre acceleration zone was marked between the approach start line and the directional change point (Dos’ Santos et al., 2021). Timing gates (SmartSpeed, Fusion Sport, Brisbane, Australia) were placed at both ends of the acceleration zone to monitor approach velocity. Participants were instructed to approach the cutting point at a controlled speed of 3.5 ± 0.2 m/s (Kim et al., 2014). A rest interval of 1 minute was provided between repeated trials of the same condition, and a 3-minute rest was given between different experimental conditions. Three successful trials were collected for each experimental condition.

SV condition was induced using specialized eyewear (Senaptec Strobe, Senaptec LLC, Portland, OR, USA). The strobe glasses were set to Level 4, producing a frequency of 3 Hz, with 0.1 seconds of clear vision followed by 0.233 seconds of occlusion (Kroll et al., 2023; Harrison et al., 2024). In the FV condition, participants performed without eyewear, maintaining an uninterrupted visual field. The ball-carrying trials followed the same experimental setup as the non-ball-carrying trials. Participants carried the rugby ball with their hands while performing the side-cutting manoeuver. The left plant foot contacted the force platform during the stance phase of the side-cutting manoeuver. During ball-carrying trials, participants carried a regulation rugby ball securely with both hands throughout the approach and side-cutting manoeuver. Trials were repeated if the ball was dropped, if secure ball possession was not maintained, or if the prescribed movement pattern was not completed. Before data collection, participants completed at least three practice trials under each task condition and were allowed additional trials until they were familiar with the protocol.

### 2.4 Data processing

Kinematic and kinetic data were processed using Visual3D software (V2025; C-Motion Inc., Germantown, MD, USA). A skeletal model and inverse-dynamics model were constructed based on the marker configuration. Mean values from the three successful trials in each condition were used for further analysis. Joint angles and velocity variables were low-pass filtered using a fourth-order Butterworth filter with a cut-off frequency of 6 Hz. GRF data were low-pass filtered using a fourth-order Butterworth filter with a cut-off frequency of 30 Hz. Joint moments and joint powers calculated using inverse dynamics were subsequently low-pass filtered using a fourth-order Butterworth filter with a cut-off frequency of 10 Hz. The IC was defined as the point at which vertical GRF exceeded 10 N, and toe-off (TO) was defined as the point when it fell below 10 N (McLean et al., 2005; Zeni Jr et al., 2008). The stance phase was defined as the interval from IC to TO. Joint moments and joint powers were calculated using inverse dynamics. GRF data were normalized to body weight and are reported in body-weight units (BW). Joint moments and joint powers were normalized to body mass and are reported in N·m·kg^−^¹ and W·kg^−^¹, respectively. All velocity variables are reported in m·s^−^¹. According to the Visual3D coordinate-system convention, the X-, Y-, and Z-axes represented the mediolateral, anterior–posterior, and vertical directions, respectively. Accordingly, F_x_, F_y_, and F_z_ represented the mediolateral, anterior–posterior, and vertical GRF components, respectively. Positive F_x_, F_y_, and F_z_ values indicated forces directed toward the positive X-, Y-, and Z-axes of the laboratory coordinate system. M_x_ represented the sagittal-plane joint moment, whereas M_y_ represented the frontal-plane joint moment. Positive and negative joint-moment values followed the right-hand-rule convention. For the left support limb, the default segment coordinate system was oriented with the X-axis directed medially, the Y-axis anteriorly, and the Z-axis superiorly.

he first peak GRF after IC was extracted for each force component. Peak joint moments and peak joint powers were extracted during the stance phase. *V*_IC_ and *V*_TO_ were defined as the instantaneous resultant velocities at IC and TO, respectively. *V*_0_ was defined as the instantaneous resultant velocity of the center of mass at its lowest vertical position during the stance phase.

The position of the COM (*r*_COM_) at time *t* was estimated as the mass-weighted average of the segmental COM positions (Equation 1). The COM velocity (*v*_COM_) was defined as the time derivative of its position. For discrete-time data sampled at constant intervals, the *v*_COM_ at frame *t_k_* was approximated using central difference (Equations 1-4). To quantify planar movement during cutting manoeuvers, horizontal COM velocity (*v*_COM,horiz_) was computed as Equation 5.

(1) $$r_{\text{COM}}\left(t\right)=\frac{1}{M}\sum_{j=1}^{n}m_{j}r_{j}^{\text{COM}}\left(t\right)$$

(2) $$v_{\text{COM}}\left(t\right)=\frac{dr_{\text{COM}}\left(t\right)}{dt}=\frac{1}{M}\sum_{j=1}^{n}m_{j}\frac{dr_{j}^{\text{COM}}\left(t\right)}{dt}$$

(3) $$v_{\text{COM}}\left(t_{k}\right)\approx \frac{1}{M}\sum_{j=1}^{n}m_{j}\cdot \frac{r_{j}^{\text{COM}}\left(t_{k+1}\right)-r_{j}^{\text{COM}}\left(t_{k-1}\right)}{2\Delta t}$$

(4) $$v_{\text{COM}}\left(t\right)=\left[\begin{array}{c} v_{x}\left(t\right)\\ v_{y}\left(t\right)\\ v_{z}\left(t\right) \end{array}\right]\in {\mathbb{R}}^{3}$$

(5) $$v_{\text{COM},\text{horiz}}\left(t\right)=\sqrt{v_{x}{\left(t\right)}^{2}+v_{y}{\left(t\right)}^{2}}$$

GRFs (*F*_GRF_) were used to quantify external loading during foot-ground interactions. At each time point *t*, the GRF vector was defined in a global three-dimensional coordinate system as Equation 6, where *F_x_*, *F_y_* and *F_z_* represent the mediolateral (side-to-side), anterior-posterior (forward-backward) and vertical components of the ground reaction force, respectively. The normalized force (*F_i_*_,norm_) was computed using Equation 7, where *m* is the subject’s body mass and *g* = 9.81 m/s^2^ is gravitational acceleration.

(6) $$F_{\text{GRF}}\left(t\right)=\left[\begin{array}{c} F_{x}\left(t\right)\\ F_{y}\left(t\right)\\ F_{z}\left(t\right) \end{array}\right]\in {\mathbb{R}}^{3}$$

(7) $$F_{i,\;\text{norm}}\left(t\right)=\frac{F_{i}\left(t\right)}{m\cdot g}\;\left(i=x,y,z\right)$$

Joint angular kinematics were derived from the relative orientation between adjacent segments. For each joint, the local rotation matrix between the distal (*R_d_*) and proximal (*R_p_*) segments was computed as Equations 8, 9.

(8) $$R_{p}\left(t\right),R_{d}\left(t\right)\in {\mathbb{R}}^{3\times 3}$$

(9) $$R_{\text{joint}}\left(t\right)=R_{p}^{⊤}\cdot R_{d}$$

This relative rotation matrix was then converted into a set of joint angles using the inverse Euler transformation:

(10) $$\theta_{\text{joint}}\left(t\right)=Euler^{-1}\left(R_{\text{joint}}\left(t\right)\right)=\left[\begin{array}{c} \theta_{1}\left(t\right)\\ \theta_{2}\left(t\right)\\ \theta_{3}\left(t\right) \end{array}\right]\in {\mathbb{R}}^{3}$$

In Equation 10, *θ*_1_, *θ*_2_ and *θ*_3_ represent the joint angles about the selected anatomical axes (e.g., flexion-extension, abduction-adduction, internal-external rotation), and the specific Euler sequence used (e.g., XYZ or ZYX) was consistent with Visual3D’s default settings.

The linear acceleration of the segmental center of mass (*a*_COM,_*_d_*) in the global coordinate system was defined as the second derivative of its position (Equation 11). The angular velocity (*ω_d_*) of the distal segment was computed from the time derivative of its rotation matrix (Equation 12). For discrete-time motion capture data, *ω_d_* was approximated using central differences applied to Euler angles (Equation 13). Angular acceleration (*α_d_*) was defined as the time derivative of *ω_d_* (Equations 14, 15). The segmental inertia tensor in the global ( $I_{d}^{\text{lab}}$) coordinate system was calculated as Equation 16. The position vector from the joint center to the segment’s COM ( $r_{\text{joint}\to \text{COM}}$) was defined as Equation 17. The net force acting on the segment (*F*_net_) was given by Equation 18. The net moment about the segment’s COM included contributions from angular acceleration and angular momentum (Equation 19). Finally, the joint moment was computed by subtracting the moment generated by the net force (due to segment COM offset) from the net moment (Equation 20).

(11) $$a_{\text{COM},d}\left(t\right)=\frac{d^{2}r_{\text{COM},d}\left(t\right)}{dt^{2}}$$

(12) $$\omega_{d}\left(t\right)=\text{Vec}\left({\left[\dot{R_{d}}\left(t\right)\cdot R_{d}^{⊤}\left(t\right)\right]}_{\times }^{-1}\right)$$

(13) $$\alpha_{d}\left(t_{k}\right)\approx \frac{\theta_{d}\left(t_{k+1}\right)-\theta_{d}\left(t_{k-1}\right)}{2\Delta t}$$

(14) $$\alpha_{d}\left(t\right)=\frac{d\omega_{d}\left(t\right)}{dt}$$

(15) $$\alpha_{d}\left(t_{k}\right)\approx \frac{\omega_{d}\left(t_{k+1}\right)-\omega_{d}\left(t_{k-1}\right)}{2\Delta t}$$

(16) $$I_{d}^{\text{lab}}\left(t\right)=R_{d}\left(t\right)\cdot I_{\text{body}}\cdot R_{d}^{⊤}\left(t\right)$$

(17) $$r_{\text{joint}\to \text{COM}}\left(t\right)=r_{\text{COM},d}\left(t\right)-r_{\text{joint}}\left(t\right)$$

(18) $$F_{\text{net}}\left(t\right)=m_{d}\cdot a_{\text{COM},d}\left(t\right)$$

(19) $$M_{\text{net}}\left(t\right)=I_{d}^{\text{lab}}\left(t\right)\cdot \alpha_{d}\left(t\right)+\omega_{d}\left(t\right)\times \left(I_{d}^{\text{lab}}\left(t\right)\cdot \omega_{d}\left(t\right)\right)$$

(20) $$M_{\text{joint}}\left(t\right)=M_{\text{net}}\left(t\right)-r_{\text{joint}\to \text{COM}}\left(t\right)\times F_{\text{net}}\left(t\right)=\left[\begin{array}{c} M_{1}\left(t\right)\\ M_{2}\left(t\right)\\ M_{3}\left(t\right) \end{array}\right]\in {\mathbb{R}}^{3}$$

Joint power (*P_joint_*) was computed as the scalar product of joint moment (*M_joint_*) and angular velocity (*ω_joint_*), providing a time-resolved indicator of mechanical work at each joint. First, the relative angular velocity of a joint was obtained by subtracting the angular velocity of the proximal segment (*ω_p_*) from that of the distal segment (*ω_d_*). Then, *P_joint_* was calculated as the dot product of the *M_joint_* and the *ω_joint_* vector (Equations 21, 22).

(21) $$\omega_{joint}\left(t\right)=\omega_{d}\left(t\right)-\omega_{p}\left(t\right)$$

(22) $$P_{joint}\left(t\right)={\left(M_{\text{joint}}\left(t\right)\right)}^{⊤}\cdot \omega_{joint}\left(t\right)=\left[\begin{array}{c} P_{1}\left(t\right)\\ P_{2}\left(t\right)\\ P_{3}\left(t\right) \end{array}\right]\in {\mathbb{R}}^{3}$$

### 2.5 Statistical analysis

All statistical analyses were performed using SPSS software (27.0, IBM, USA). Data were first assessed for normality using the Shapiro–Wilk test. For variables that met the assumption of normal distribution, paired-sample t-tests were conducted to compare outcomes between the FV and SV conditions. For non-normally distributed data, the paired-sample Wilcoxon signed-rank test was employed. Effect sizes for pairwise comparisons were calculated using Cohen’s d, where values were interpreted as trivial (< 0.20), small (0.20–0.49), medium (0.50–0.79), and large (≥ 0.80) (Cohen, 2013). Effect sizes for pairwise comparisons were calculated as $r=\left|Z\right|/\sqrt{N}$, where *Z* is the standardized Wilcoxon signed-rank test statistic and *N* is the number of paired observations. Effect sizes r were interpreted as trivial (< 0.10), small (0.10–0.29), moderate (0.30–0.49), and large (≥ 0.50). (Cohen, 2013). The alpha level for statistical significance was set at p< 0.05. Bonferroni-adjusted p values were calculated by multiplying each unadjusted p value by three, with adjusted p values capped at 1.00 (Armstrong, 2014).

## 3 Results

### 3.1 Non-ball-carrying side-cutting manoeuver

**Figure 3:**
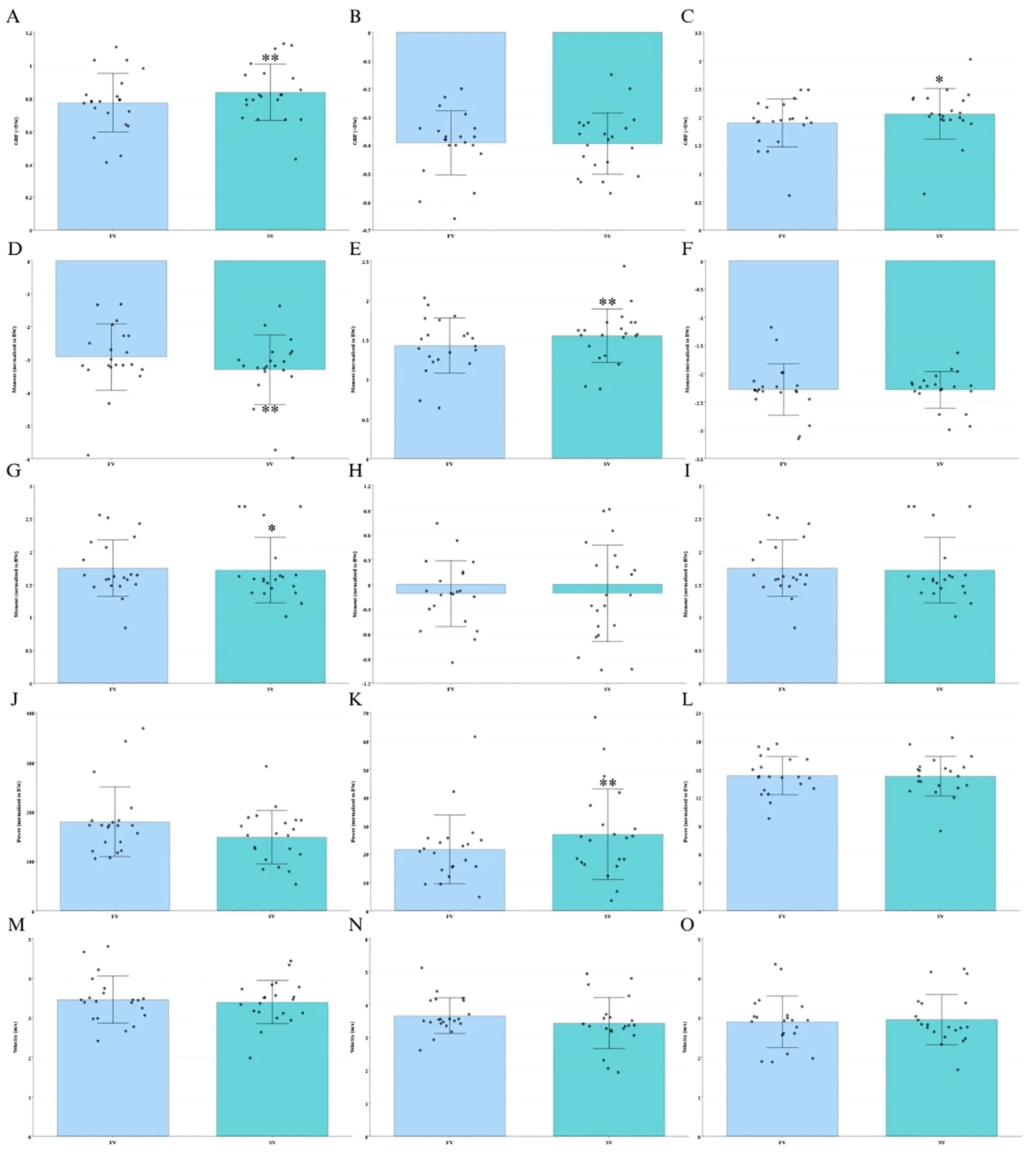
Lower-limb biomechanical variables during non-ball-carrying side-cutting under full vision and stroboscopic vision conditions. **(A)** Fx; **(B)** Fy; **(C)** Fz; **(D)** hip Mx; **(E)** knee Mx; **(F)** ankle Mx; **(G)** hip My; **(H)** knee My; **(I)** ankle My; **(J)** hip power; **(K)** knee power; **(L)** ankle power; **(M)** *V*_IC_; **(N)** *V*_TO_; and **(O)** *V*_0_; *p Bonferroni< 0.05, **p Bonferroni< 0.01.

The results of the non-ball-carrying side-cutting manoeuvers are presented in **Table 2** and **Figure 3**. Under the SV condition, F_x_ significantly increased compared with the FV condition (p = 0.001, d = 0.877), indicating a large effect size. Similarly, F_z_ was significantly greater under SV (p = 0.011, d = 0.614), with a moderate effect size. No significant difference was observed in F_y_ (p = 0.099, d = 0.378). For joint moments, M_x_ at the hip was significantly more negative under SV than under FV (p< 0.001, r = 0.808), whereas M_x_ at the knee was significantly greater under SV (p = 0.002, r = 0.693); both effects were large. No significant difference was observed in ankle M_x_ (p = 0.384, r = 0.190). In the M_y_ direction, hip moment was significantly more negative under SV (p = 0.010, r = 0.565), representing a large effect, whereas no significant differences were found at the knee (p = 0.096, r = 0.368) or ankle (p = 0.348, r = 0.205). Knee joint power was significantly greater under SV than under FV (p< 0.001, r = 0.869), indicating a large effect size. No significant differences were observed in hip power (p = 0.060, r = 0.413) or ankle power (p = 0.801, d = 0.056). In addition, V_ic_, V_to_, and V_0_ did not differ significantly between visual conditions (all p > 0.05).

**Table 2:** Lower-limb kinetic and kinematic variables during non-ball-carrying side-cutting manoeuvers.

Variable	FV	SV	t/z	p	d/r
F_x_ (BW)	0.80 ± 0.11	0.90 ± 0.12	t = -4.02	0.001^**^	d = 0.877
F_y_ (BW)	-0.42 ± 0.11	-0.37 ± 0.14	t = -1.732	0.099	d = 0.378
F_z_ (BW)	2.07 ± 0.30	2.27 ± 0.40	t = -2.813	0.011^*^	d = 0.614
M_x Hip_ (N·m·kg^−^¹)	-3.340(-3.6,-3.0)	-3.450(-3.8,-3.3)	z = 3.703	<0.001^**^	r = 0.808
M_x Knee_ (N·m·kg^−^¹)	1.310(1.1,1.4)	1.400(1.2,1.6)	z = -3.174	0.002^**^	r = 0.693
M_x Ankle_ (N·m·kg^−^¹)	-2.210(-2.3,-2.1)	-2.200(-2.3,-1.9)	z = -0.87	0.384	r = 0.190
M_y Hip_ (N·m·kg^−^¹)	-0.830(-1.3,-0.7)	-0.980(-1.3,-0.9)	z = 2.591	0.010^**^	r = 0.565
M_y Knee_ (N·m·kg^−^¹)	0.120(-0.5,0.2)	0.190(-0.2,0.6)	z = -1.686	0.096	r = 0.368
M_y Ankle_ (N·m·kg^−^¹)	1.810(1.7,2.0)	1.770(1.5,2.0)	z = 0.939	0.348	r = 0.205
P _Hip_ (W·kg^−^¹)	168.590(139.6,179.5)	182.470(138.1,192.7)	z = -1.894	0.060	r = 0.413
P _Knee_ (W·kg^−^¹)	14.740(7.2,16.4)	17.320(14.7,21.0)	z = -3.98	<0.001^**^	r = 0.869
P _Ankle_ (W·kg^−^¹)	14.61 ± 2.01	14.72 ± 1.99	t = -0.255	0.801	d = 0.056
V _IC_ (m·s^−^¹)	3.46 ± 0.60	3.39 ± 0.54	t = 0.526	0.605	d = 0.115
V _TO_ (m·s^−^¹)	3.66 ± 0.54	3.43 ± 0.77	t = 1.739	0.097	d = 0.380
V_0_ (m·s^−^¹)	2.89 ± 0.65	2.95 ± 0.64	t = -0.657	0.519	d = 0.143

The results of paired-samples t-tests are presented as mean ± SD, whereas the results of Wilcoxon signed-rank tests are presented as median (P25, P75). FV, full vision condition; SV, stroboscopic vision condition; V, instantaneous velocity; F, peak ground reaction force; M, peak joint moment; P, peak joint power. d, Cohen’s d; r, effect size calculated as $\left|\text{Z}\right|/\sqrt{\text{N}}$ for the Wilcoxon signed-rank test. Cohen’s d was interpreted as trivial (< 0.20), small (0.20–0.49), moderate (0.50–0.79), and large (≥ 0.80). Effect size r was interpreted as trivial (< 0.10), small (0.10–0.29), moderate (0.30–0.49), and large (≥ 0.50).

The symbols indicate statistically significant differences between conditions: *p < 0.05 and **p < 0.01

### 3.2 Ball-carrying side-cutting manoeuver

**Figure 4:**
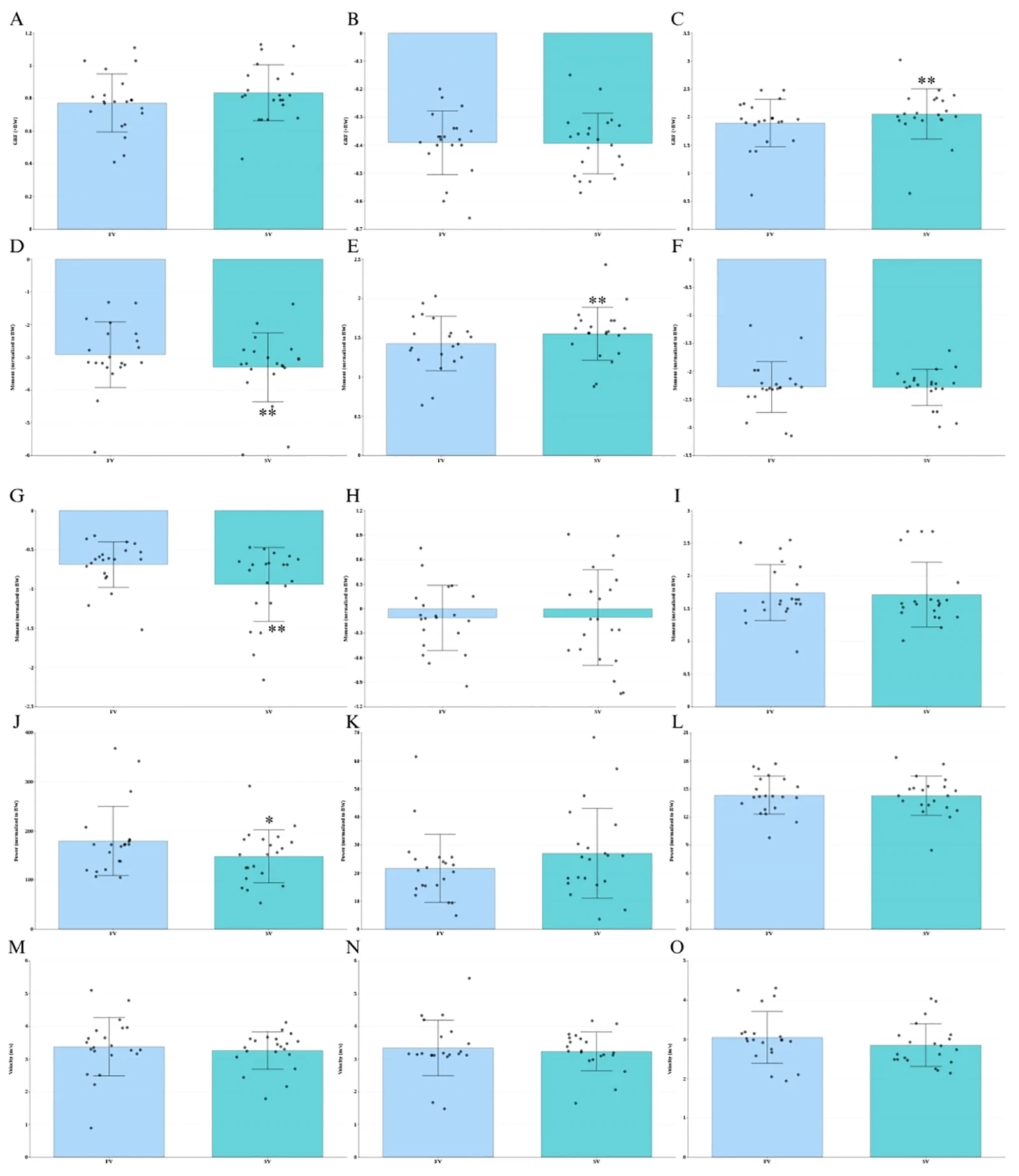
Lower-limb biomechanical variables during ball-carrying side-cutting under full vision and stroboscopic vision conditions. **(A)** Fx; **(B)** Fy; **(C)** Fz; **(D)** hip Mx; **(E)** knee Mx; **(F)** ankle Mx; **(G)** hip My; **(H)** knee My; **(I)** ankle My; **(J)** hip power; **(K)** knee power; **(L)** ankle power; **(M)** *V*_IC_; **(N)** *V*_TO_; and **(O)** *V*_0_; *p Bonferroni< 0.05, **p Bonferroni< 0.01.

The results of the ball-carrying side-cutting manoeuver are presented in **Table 3** and **Figure 4**. Under the SV condition, F_x_ significantly increased compared with the FV condition (p = 0.020, d = 0.549), indicating a moderate effect size. F_z_ was also significantly greater under SV (p = 0.001, r = 0.726), representing a large effect size. No significant difference was observed in F_y_ (p = 0.923, d = 0.021). For joint moments, hip M_x_ was significantly more negative under SV than under FV (p< 0.001, r = 0.876), representing a large effect size. Knee M_x_ was significantly greater under SV (p = 0.002, d = 0.785), with a moderate effect size, whereas no significant difference was observed in ankle M_x_ (p = 0.639, r = 0.102). In the M_y_ direction, hip moment was significantly more negative under SV (p = 0.001, r = 0.747), indicating a large effect size. No significant differences were found in knee M_y_ (p = 0.972, d = 0.008) or ankle M_y_ (p = 0.681, r = 0.090). Regarding peak joint power, knee power was significantly greater under SV than under FV (p = 0.016, r = 0.527), representing a large effect size. However, no significant differences were observed in hip power (p = 0.229, r = 0.269) or ankle power (p = 0.877, d = 0.034). V _IC_ and V _TO_ did not differ significantly between visual conditions (both p > 0.05). However, V_0_ was significantly lower under SV than under FV (p = 0.025, r = 0.489), indicating a moderate effect size.

**Table 3:** Lower-limb kinetic and kinematic variables during ball-carrying side-cutting manoeuvers.

Variable	FV	SV	t/z	p	d/r
F_x_ (BW)	0.77 ± 0.18	0.84 ± 0.17	t = -2.518	0.020^*^	d = 0.549
F_y_ (BW)	-0.39 ± 0.11	-0.39 ± 0.11	t = 0.098	0.923	d = 0.021
F_z_ (BW)	1.940(1.9,2.2)	2.040(1.9,2.3)	z = -3.326	0.001^**^	r = 0.726
M_x Hip_ (N·m·kg^−^¹)	-3.150(-3.2,-2.3)	-3.190(-3.4,-2.8)	z = 4.015	<0.001^**^	r = 0.876
M_x Knee_ (N·m·kg^−^¹)	1.43 ± 0.35	1.55 ± 0.34	t = -3.596	0.002^**^	d = 0.785
M_x Ankle_ (N·m·kg^−^¹)	-2.290(-2.3,-2.2)	-2.230(-2.3,-2.2)	z = -0.469	0.639	r = 0.102
M_y Hip_ (N·m·kg^−^¹)	-0.620(-0.8,-0.5)	-0.690(-1.2,-0.7)	z = 3.424	0.001^**^	r = 0.747
M_y Knee_ (N·m·kg^−^¹)	-0.11 ± 0.40	-0.11 ± 0.58	t = -0.036	0.972	d = 0.008
M_y Ankle_ (N·m·kg^−^¹)	1.620(1.5,2.1)	1.580(1.4,1.6)	z = 0.411	0.681	r = 0.09
P _Hip_ (W·kg^−^¹)	172.210(138.4,182.0)	152.160(114.0,182.8)	z = 1.234	0.229	r = 0.269
P _Knee_ (W·kg^−^¹)	20.970(15.4,24.9)	25.720(17.1,30.4)	z = -2.416	0.016^*^	r = 0.527
P _Ankle_ (W·kg^−^¹)	14.34 ± 2.03	14.28 ± 2.10	t = 0.156	0.877	d = 0.034
V _IC_ (m·s^−^¹)	3.350(3.2,3.9)	3.380(3.1,3.6)	z = -0.191	0.848	r = 0.042
V _TO_ (m·s^−^¹)	3.160(3.1,3.7)	3.240(3.1,3.6)	z = 0.904	0.366	r = 0.197
V_0_ (m·s^−^¹)	2.990(2.8,3.1)	2.740(2.5,3.1)	z = 2.242	0.025^*^	r = 0.489

The results of paired-samples t-tests are presented as mean ± SD, whereas the results of Wilcoxon signed-rank tests are presented as median (P25, P75). FV: full vision condition; SV: stroboscopic vision condition; V, instantaneous velocity; F, peak ground reaction force; M, peak joint moment; P, peak joint power. d, Cohen’s d; r, effect size calculated as $\left|\text{Z}\right|/\sqrt{\text{N}}$ for the Wilcoxon signed-rank test. Cohen’s d was interpreted as trivial (< 0.20), small (0.20–0.49), moderate (0.50–0.79), and large (≥ 0.80). Effect size r was interpreted as trivial (< 0.10), small (0.10–0.29), moderate (0.30–0.49), and large (≥ 0.50).

The symbols indicate statistically significant differences between conditions: *p < 0.05 and **p < 0.01

### 3.3 Bonferroni-adjusted results

Bonferroni-adjusted p values are presented in **Table 4**. Following Bonferroni correction, all statistically significant findings observed during non-ball-carrying side-cutting remained significant, including F_x_ ( ${\text{p}}_{\text{Bonferroni}}=0.003$), F_z_ ( ${\text{p}}_{\text{Bonferroni}}=0.033$), hip M_x_ ( ${\text{p}}_{\text{Bonferroni}}<0.001$), knee M_x_ ( ${\text{p}}_{\text{Bonferroni}}=0.006$), hip M_y_ ( ${\text{p}}_{\text{Bonferroni}}=0.030$), and peak knee joint power ( ${\text{p}}_{\text{Bonferroni}}<0.001$). During ball-carrying side-cutting, significant differences in F_z_ ( ${\text{p}}_{\text{Bonferroni}}=0.003$), hip M_x_ ( ${\text{p}}_{\text{Bonferroni}}<0.001$), knee M_x_ ( ${\text{p}}_{\text{Bonferroni}}=0.006$), hip M_y_ ( ${\text{p}}_{\text{Bonferroni}}=0.003$), and peak knee joint power ( ${\text{p}}_{\text{Bonferroni}}=0.048$) remained after correction. In contrast, the unadjusted differences in F_x_ ( p=0.020) and *V*_0_ ( p=0.025) were no longer statistically significant after Bonferroni adjustment ( ${\text{p}}_{\text{Bonferroni}}=0.060$ and 0.075, respectively).

**Table 4:** Unadjusted and Bonferroni-adjusted p values for statistically significant task-specific comparisons between full vision and stroboscopic vision conditions.

Variable	p Unadjusted	p Bonferroni
Non-ball		
F_x_ (BW)	0.001	0.003^**^
F_z_ (BW)	0.011	0.033^*^
M_x Hip_ (N·m·kg^−^¹)	<0.001	<0.001^**^
M_x Knee_ (N·m·kg^−^¹)	0.002	0.006^**^
M_y Hip_ (N·m·kg^−^¹)	0.010	0.030^*^
P _Knee_ (W·kg^−^¹)	<0.001	<0.001^**^
Ball-carrying		
F_x_ (BW)	0.020	0.060
F_z_ (BW)	0.001	0.003^**^
M_x Hip_ (N·m·kg^−^¹)	<0.001	<0.001^**^
M_x Knee_ (N·m·kg^−^¹)	0.002	0.006^**^
M_y Hip_ (N·m·kg^−^¹)	0.001	0.003^**^
P _Knee_ (W·kg^−^¹)	0.016	0.048^*^
V_0_ (m·s^−^¹)	0.025	0.075

*p Bonferroni< 0.05, **p Bonferroni< 0.01.

## 4 Discussion

This study examined the acute effects of SV on lower-limb kinetics and joint mechanics during rugby-related side-cutting manoeuvers performed with and without ball-carrying. After Bonferroni adjustment, SV significantly increased F_x_ and F_z_ during non-ball-carrying side-cutting, whereas, during ball-carrying side-cutting, only F_z_ remained significantly greater under SV. F_y_ was unchanged in both task conditions. At the joint level, hip M_x_ became more negative and knee M_x_ increased under SV in both task conditions, whereas ankle M_x_ was unaffected. Hip M_y_ was also more negative under SV in both non-ball-carrying and ball-carrying trials, while no significant changes were observed in knee or ankle M_y_. Peak knee joint power was greater under SV in both task conditions, whereas hip and ankle joint power did not differ significantly between visual conditions. Collectively, these findings indicate that the biomechanical effects of acute SV exposure were most consistently observed in vertical loading and hip and knee joint mechanics, with no detectable changes in ankle joint kinetics.

The acute exposure to SV elicited pronounced changes in lower-limb kinetics and joint mechanics, indicating that intermittent visual occlusion was associated with altered lower-limb kinetics and joint mechanics during side-cutting. The significant increases in mediolateral and vertical GRF under SV indicate greater peak mediolateral and vertical loading during direction change (Kroll et al., 2023). These heightened forces likely arise from the CNS’s attempt to preserve dynamic balance and trajectory accuracy through increased muscular co-contraction and joint stiffness (Latash, 2018; Koo et al., 2023); however, these variables were not directly assessed. The SV-related changes in hip and knee joint moments about the M_x_ further support this interpretation: hip M_x_ became more negative, whereas knee M_x_ increased under SV., suggesting that he biomechanical effects of SV were more evident at the hip and knee than at the ankle (Nataraj et al., 2023). Comparable kinetic adaptations have been reported in plyometric contexts, where SV exposure increased vertical GRF and knee joint loading during depth jumps. Kroll et al. (2023) observed that female Division I volleyball athletes exhibited greater braking impulses and altered landing mechanics under SV, suggesting an altered landing strategy under intermittent visual occlusion (Kroll et al., 2023). Similarly, Harrison et al. (2024) demonstrated that SV heightened joint moments and reduced post-landing variability, which has been interpreted as a possible compensatory response to intermittent visual occlusion (Harrison et al., 2024). These findings across jumping and cutting tasks suggest that acute SV exposure may be associated with altered lower-limb loading and joint-moment patterns under conditions of intermittent visual occlusion.

The mechanical alterations observed under SV may be discussed in relation to the theoretical framework of sensory reweighting, a compensatory process through which the CNS may adjust the relative reliance on visual, vestibular, and proprioceptive inputs when visual information becomes less reliable (Peterka, 2002; Assländer and Peterka, 2014). Previous balance and locomotor research suggests that intermittent or less reliable visual information can be accompanied by a greater relative reliance on somatosensory and vestibular cues (Pasma et al., 2015). This adaptive redistribution has been demonstrated in balance and locomotor tasks where reduced visual confidence promotes greater dependence on proprioceptive feedback for postural control (Peterka, 2002). In the present context, the increased peak F_x_ and F_z_, together with the SV-related changes in hip and knee moments about the M_x_, may be compatible with altered force-regulation strategies under intermittent visual occlusion. During ball-carrying trials, the lower V_0_ observed under SV may be compatible with a more conservative movement strategy in the presence of intermittent visual occlusion (Stokes et al., 2017; Richards et al., 2019). Similar cautious movement patterns have been reported in visually perturbed gait and landing studies; however, the present data do not allow direct identification of the underlying control strategy (Richards et al., 2019). Within the present cutting paradigm, this deceleration pattern reflects a shift from predictive, feed-forward control toward greater feedback-driven stabilization (Gordon et al., 2020; Ijspeert and Daley, 2023). Hip moment about the M_y_ was more negative under SV in both non-ball-carrying and ball-carrying trials, indicating that the effect of SV on hip frontal-plane kinetics was not limited to the ball-carrying condition. However, cortical activity and attentional allocation were not measured in the present study. Consequently, the observed changes in joint kinetics and segmental coordination may reflect altered biomechanical responses to intermittent visual occlusion, but the neural and coordinative mechanisms underlying these changes require direct investigation. These findings show that acute SV exposure alters lower-limb biomechanical variables during side-cutting. These changes may be discussed in relation to sensory–motor adaptation (Dewan et al., 2023; Clark, 2024). Whether repeated exposure to SV produces long-term perceptual–motor or biomechanical adaptations remains unknown and should be examined in longitudinal studies incorporating direct neuromuscular, perceptual, and performance measures. The acute biomechanical responses observed under SV offer important insights into the potential mechanisms underpinning stroboscopic training. The acute increases in peak F_x_ and F_z_, together with the SV-related changes in hip and knee joint moments collectively suggest that SV imposes a controlled form of sensorimotor stress—one that challenges the stability of perception–action coupling without altering task structure (Wilkins and Appelbaum, 2020; Jothi et al., 2025). It is possible that repeated exposure to visual perturbations may contribute to training adaptations; however, whether SV training alters sensory weighting or improves movement efficiency requires confirmation in longitudinal intervention studies (Zemková and Kováčiková, 2023; Appiah-Kubi et al., 2024; Ketterer et al., 2024). This aligns with the concept of perceptual–motor robustness, wherein performance stability is preserved across variable sensory contexts. By intermittently disrupting visual feedback, repeated SV exposure may potentially challenge feed-forward movement regulation and proprioceptive–vestibular integration (Beck et al., 2023; Vasile and Stănescu, 2024). However, whether SV training alters these sensory–motor processes remains to be established through longitudinal studies incorporating direct neuromuscular and neurophysiological measures. Over time, repeated SV exposure may potentially influence neural efficiency and reliance on continuous visual monitoring (Li and Smith, 2021; Shi et al., 2024). These outcomes were not assessed in the present study and require confirmation through longitudinal studies incorporating direct neurophysiological and perceptual measures. Such processes may be relevant to open-skill sports such as rugby, in which visual attention is dynamically allocated among ball trajectory, opponent movement, and available space. Moreover, the finding that the effects of SV were more evident at the hip and knee joints than at the ankle, as no significant changes were observed in ankle joint moments or power. Future studies should determine whether repeated SV training influences multi-joint movement strategies relevant to cutting, tackling, and aerial contests, as well as their implications for movement safety and sport-specific performance (Kim et al., 2017). In practice, integrating SV exposure into agility or reactive decision-making drills could serve as an advanced stimulus for enhancing contextual anticipation, postural resilience, and movement adaptability under perceptual uncertainty (Li et al., 2024b; Vasile and Stănescu, 2024; Zwierko et al., 2024b). Recent studies have already demonstrated improved passing accuracy, decision speed, and spatial awareness following SV interventions in team sport contexts, supporting the notion that acute biomechanical adjustments such as those observed here may form the foundation of long-term perceptual–motor learning (Wilkins and Gray, 2015; Zwierko et al., 2024a). Although some intervention studies have reported improvements in selected perceptual–motor outcomes following SV training, it remains uncertain whether the acute biomechanical changes observed in the present study contribute to long-term learning or performance adaptation. Future research may compare SV with other task constraints to determine how different training stimuli influence movement biomechanics and sport-specific performance (Afán-Argüín et al., 2023; Das et al., 2023; Wohl et al., 2024; Lu et al., 2025). S Whether adaptations induced by SV-based training transfer to competitive rugby performance remains to be established (Assländer and Peterka, 2016; Hülsdünker et al., 2021).

Several limitations of the present study should be acknowledged when interpreting the findings. First, the investigation examined only the acute effects of SV exposure within a single testing session. Although this design effectively isolated the immediate biomechanical adjustments to intermittent visual occlusion, it did not capture potential long-term adaptations that may arise from repeated SV training. Future longitudinal studies should therefore examine whether the transient increases in braking force, joint torque, and sensory reweighting observed here evolve into persistent improvements in perceptual–motor robustness or agility performance. Second, the experimental paradigm focused primarily on kinetic and kinematic variables. The absence of concurrent neurophysiological and muscle activation data—such as surface electromyography (sEMG), electroencephalography (EEG), or functional near-infrared spectroscopy (fNIRS)—limits the ability to directly link mechanical outcomes with neural or sensory-level mechanisms. Incorporating multimodal signal acquisition in future work would enable a more comprehensive understanding of the cortical, subcortical, and spinal contributions to SV-induced motor control adaptations. Individual variability in visual–cognitive capacity and prior perceptual training experience may modulate the magnitude of response to SV exposure. Future studies should adopt stratified designs or machine-learning approaches to model how baseline sensory profiles predict adaptation trajectories. Such work could inform personalized stroboscopic training protocols, optimizing the frequency, duration, and task context for different athlete populations. Despite these limitations, the current findings establish a mechanistic foundation for understanding how intermittent visual disruption acutely alters lower-limb biomechanics and control strategies during complex movements. Extending this research through longitudinal, multimodal, and ecologically valid approaches will help delineate how SV exposure can be systematically harnessed to enhance perceptual–motor adaptability, stability, and performance in high-intensity, visually demanding sports such as rugby.

## 5 Conclusion

This study examined the acute effects of SV on lower-limb biomechanics during rugby-related side-cutting manoeuvers performed with and without ball-carrying. Following Bonferroni adjustment, SV increased peak F_x_ and F_z_ during non-ball-carrying side-cutting, whereas only F_z_ remained significantly greater during ball-carrying side-cutting. Across both task conditions, SV was associated with more negative hip M_x_ and M_y_, greater knee M_x_, and greater peak knee joint power, while ankle joint moments and power were unchanged. Overall, acute SV exposure was associated with altered lower-limb loading and joint mechanics, with the most consistent effects observed at the hip and knee joints. Future longitudinal studies incorporating neuromuscular, perceptual, and performance measures are needed to determine whether repeated SV exposure produces adaptations relevant to rugby performance.

## 6 Practical applications

Acute SV exposure may be used as a controlled visual perturbation when investigating lower-limb biomechanics during rugby-related side-cutting. In the present study, SV was associated with altered peak ground reaction forces and hip and knee joint kinetics, suggesting that intermittent visual occlusion can modify movement mechanics during directional-change tasks. However, the present findings do not establish training benefits, improved decision-making, enhanced postural control, or reduced injury risk. Future longitudinal studies should evaluate the feasibility, safety, optimal dosage, and performance effects of incorporating SV into rugby-specific training drills.

## Data Availability

The original contributions presented in the study are included in the article/Supplementary Material. Further inquiries can be directed to the corresponding authors.
